# Rare compound heterozygous variants of *LAMB3* and histological features of enamel and oral mucosa

**DOI:** 10.3389/fphys.2022.1006980

**Published:** 2022-10-10

**Authors:** Fang Li, Miao Yu, Zhuangzhuang Fan, Junyi Wu, Hua Tian, Hailan Feng, Yang Liu, Haochen Liu, Dong Han

**Affiliations:** ^1^ Third Clinical Division, Peking University School and Hospital of Stomatology and National Center of Stomatology and National Clinical Research Center for Oral Diseases and National Engineering Research Center of Oral Biomaterials and Digital Medical Devices, Beijing, China; ^2^ Department of Prosthodontics, Peking University School and Hospital of Stomatology and National Center of Stomatology and National Clinical Research Center for Oral Diseases and National Engineering Research Center of Oral Biomaterials and Digital Medical Devices, Beijing, China; ^3^ Department of Cariology and Endodontology, Peking University School and Hospital of Stomatology and National Center of Stomatology and National Clinical Research Center for Oral Diseases and National Engineering Research Center of Oral Biomaterials and Digital Medical Devices, Beijing, China

**Keywords:** junctional epidermolysis bullosa, amelogenesis imperfecta, *LAMB3*, whole-exome sequencing, oral mucosa

## Abstract

Junctional epidermolysis bullosa (JEB) is a group of autosomal recessive disorders characterized by amelogenesis imperfecta (AI) and fragility of the skin and mucous membranes. The purpose of this study was to identify pathogenic gene variants and investigate the phenotypic characteristics of abnormal enamel structure and mucocutaneous lesions in a patient with JEB. Clinical examination of the patient revealed hypoplastic AI, skin lesions, and oral ulcers, whereas her parents were normal. Whole-exome sequencing (WES) and cDNA cloning identified compound heterozygous variants of *LAMB3* in the proband: c.125G>C in exon 3, c.1288 + 1G>A in intron 11, and c.1348C>T in exon 12. Among these, c.125G>C was inherited from her father, and the other two variants were inherited from her mother. Functional prediction indicated that the variants might change protein structure and cause disease. Scanning electron microscopy (SEM) examination of the primary and permanent teeth revealed abnormal enamel morphology and microstructures. Hematoxylin-eosin (HE) and immunofluorescence (IF) staining showed significantly abnormal and disorganized epithelial cells in the gingival mucosa. Our results showed that this was a case of intermediate JEB1A (OMIM #226650) with autosomal recessive inheritance. The proband carried rare compound heterozygous variants of *LAMB3*. Our results broaden the variant spectrum of the *LAMB3* gene and JEB cases. Moreover, this is the first study to identify histological malformations of the primary teeth and oral mucosa in *LAMB3*-related patients.

## 1 Introduction

Junctional epidermolysis bullosa (JEB) is a group of autosomal recessive disorders characterized by amelogenesis imperfecta (AI) and fragility of the skin and mucous membranes (Kim et al., 2013; Pfendner and Lucky, 2008). Broad classification of JEB includes severe JEB1B (OMIM #226700) and intermediate JEB1A (OMIM #226650) (Pfendner and Lucky, 2008). Oral manifestations of JEB include AI and significant oral and mucosal involvement. AI is a group of hereditary developmental diseases that affects the structure and clinical appearance of enamel (Crawford et al., 2007). It affects both primary and permanent dentition and can be classified into four subtypes: hypoplastic, hypo-maturation, hypocalcified, and hypo-maturation/hypoplastic with taurodontism (Witkop, 1988).

Laminin Subunit Beta 3 (LAMB3) is one of three subunits of the basement membrane protein laminin-332 (formerly laminin-5). Variants of genes in this family are associated with the inherited skin disorder junctional epidermolysis bullosa (JEB) (Nakano et al., 2002). Mice with homozygous recessive mutations in *Lamb3* showed extensive skin blisters and died within 24 h after birth (Kuster et al., 1997). In humans, biallelic mutations of *LAMB3* lead to JEB characterized by increased skin fragility; phenotype severity ranges from mild to fatal, and some patients may also exhibit enamel defects (Fine et al., 2014). When a single heterozygous variant occurs, the carrier usually has few or no apparent skin lesions and only shows dental anomalies manifested as hypoplastic AI (Kim et al., 2013; Kim et al., 2019; Kim et al., 2016; Lee et al., 2015; Poulter et al., 2014; Prasad et al., 2016; Smith et al., 2019; Wang et al., 2015). Previous studies mostly focused on phenotype observation and mutation detection, while others investigated the histological features of the skin in *LAMB3*-related JEB cases (Kiritsi et al., 2015; Mayer et al., 2016) or the microstructure of the third molar in *LAMB3*-related AI cases (Smith et al., 2019). However, there are no reports of histological examination of the oral mucosa and primary teeth in *LAMB3*-related cases.

In this study, we employed whole-exome sequencing (WES) to identify the pathogenic variants in an intermediate JEB patient with hypoplastic AI. Furthermore, we performed histological examination of teeth and oral mucosa to investigate the histological changes.

## 2 Materials and methods

### 2.1 Participants

A 20-year-old Chinese female visited the Department of Prosthodontics at the Stomatology Hospital of Peking University with a complaint of yellow and rough teeth. Her parents had normal dentition. A detailed clinical examination of the oral cavity and the skin was performed. Genotypic and histomorphological analyses were performed to explore the genetic and histological characteristics of the disease.

The study was approved by the Ethics Committee of Peking University School and Hospital of Stomatology (PKUSSIRB-201736082) and was performed in accordance with the principles of the Declaration of Helsinki. Informed consent was obtained from all family members.

### 2.2 Genotype analyses

#### 2.2.1 Whole-exome sequencing and variant analysis

Blood samples were collected from the proband and her parents, and genomic DNA was isolated using a Biotek DNA Minikit (Biotek, Beijing, China) according to the manufacturer’s instructions. Genomic DNA samples of the proband were sent for WES and copy-number variation array analysis using the Illumina X10 sequencing platform (iGeneTech, Beijing, China). The detected variants were filtered according to the following strategy: firstly, we analyzed all genes related to tooth development (Prasad et al., 2016). Secondly, we excluded nonsynonymous single-nucleotide variants and variants with a minor allele frequency (MAF) ≥0.01 in the Exome Aggregation Consortium (ExAC, http://exac.broadinstitute.org (1 June 2020), Genome Aggregation Database [gnomAD, http://gnomad.broadinstitute.org/(1 June 2020)], 1000 Genomes Project data in Ensembl [http://asia.ensembl.org/Homo_sapiens/Info/Index (1 June 2020)], and Single-Nucleotide Polymorphism database [dbSNP, http://www.ncbi.nlm.nih.gov/projects/SNP/snp_summary.cgi/(1 June 2020)]. Lastly, to confirm the variants and to analyze familial co-segregation, the coding exons and intron–exon boundaries of human *LAMB3* (NM_000228.3) were amplified by polymerase chain reaction (PCR; primer sequences are shown in Supplementary Table S1). The PCR products were sequenced by Tsingke Biological (Beijing, China), and the results were blasted against NCBI.

#### 2.2.2 RNA isolation and cDNA sequencing

The patient’s gingiva was obtained and stored in RNAlater (Thermo Fisher Scientific, Waltham, MA, United States), and total RNA was extracted using TRIzol^®^ reagent (Life Technologies, Carlsbad, CA, United States) according to the manufacturer’s instructions. Next, 100–1,000 ng of total RNA was reverse-transcribed using the Superscript First-Strand Synthesis System (Life Technologies, Carlsbad, CA, United States) (Zeng et al., 2018). Primers were designed using the Primer 3 software [http://primer3.ut.ee/(1 June 2020); primer sequences are shown in Supplementary Table S1]. The exon 10–12 fragment of *LAMB3* cDNA was amplified, and the PCR product was sequenced by Tsingke Biological.

#### 2.2.3 Conservation analysis and prediction of the variant effect

Alignment analysis of the *LAMB3* amino-acid sequence among multiple species was performed using ClustalX 2.1. Bioinformatic analyses of missense variants were conducted using PolyPhen2 [http://genetics.bwh.harvard.edu/pph2/(1 June 2020)], PROVEAN [http://provean.jcvi.org/index.php (1 June 2020)], and MutationTaster [http://www.mutationtaster.org/(1 June 2020)]. The tertiary structural changes of the LAMB3 protein were analyzed using the PyMOL Molecular Graphics System [DeLano Scientific, Palo Alto, CA, United States; http://www.pymol.org (1 June 2020)]. The pathogenicity of the three variants was classified according to the American College of Medical Genetics and Genomics (ACMG) guideline (Richards et al., 2015).

### 2.3 Histomorphology analyses of the affected teeth and mucosa

#### 2.3.1 Scanning electron microscopy examination of the affected teeth

The patient’s teeth at positions 63, 12, 26, and 32 were extracted because of mobility or severe defects. Normal teeth of the same type with an intact crown shape and a normal enamel structure were collected. After extraction, the teeth were fixed in a neutral formaldehyde solution.

The teeth were separated to expose their inner surface. The sections were then etched with 30% phosphate gel, rinsed thoroughly with distilled water, and dried overnight in a vacuum drying oven at 37°C. After coating with gold, tooth samples were observed with SEM (HITACHI SU8010; HITACHI, Tokyo, Japan) (Li et al., 2018; Li et al., 2015).

#### 2.3.2 Hematoxylin–eosin staining of the Patient’s gingival mucosa

A small piece of keratinized oral gingival mucosa in the edentulous alveolar ridge was obtained during implant surgery. Another piece of oral gingival mucosa in similar location from a subject with normal enamel and mucocutaneous presentation was collected. The sample was fixed in neutral formaldehyde solution at room temperature for 24 h, and was then embedded in paraffin, mounted on a microtome, and sectioned to a thickness of 5 μm. The slices were stained with hematoxylin and eosin following a routine process and observed under an OLYMPUS BX51 microscope (OLYMPUS, Tokyo, Japan).

#### 2.3.3 Immunofluorescence staining of the Patient’s gingival mucosa

For immunofluorescent staining, the prepared gingival epithelium sections were treated with antigen retrieval in a microwave oven, blocked with 10% normal goat serum and then incubated with anti-Keratin 5 (Biolegend-905503, 1/50), anti-Keratin 10 (Biolegend-905403, 1/50), anti-PCNA (10205-2-AP, 1/100) at 4°C overnight, followed by incubation with Alexa Fluor 594-conjugated goat anti-Rabbit IgG (ZSGB-BIO, ZF-0516) or Alexa Fluor 488-conjugated goat anti-Rabbit IgG (ZSGB-BIO, ZF-0516) at room temperature for 1 h. Subsequently, sections were mounted using mounting medium with DAPI (ZSGB-BIO, ZLI-9557) and captured by a fluorescence microscope. Evaluation of epidermal differentiation markers were carried out using ImageJ. The keratin 10 (K10) were used for evaluating the stratum spinosum cells, keratin 5 (K5) were used for evaluating the basal cells, and proliferating cell nuclear antigen (PCNA) were used for detecting the cell proliferation.

## 3 Results

### 3.1 Clinical manifestation

**FIGURE 1:**
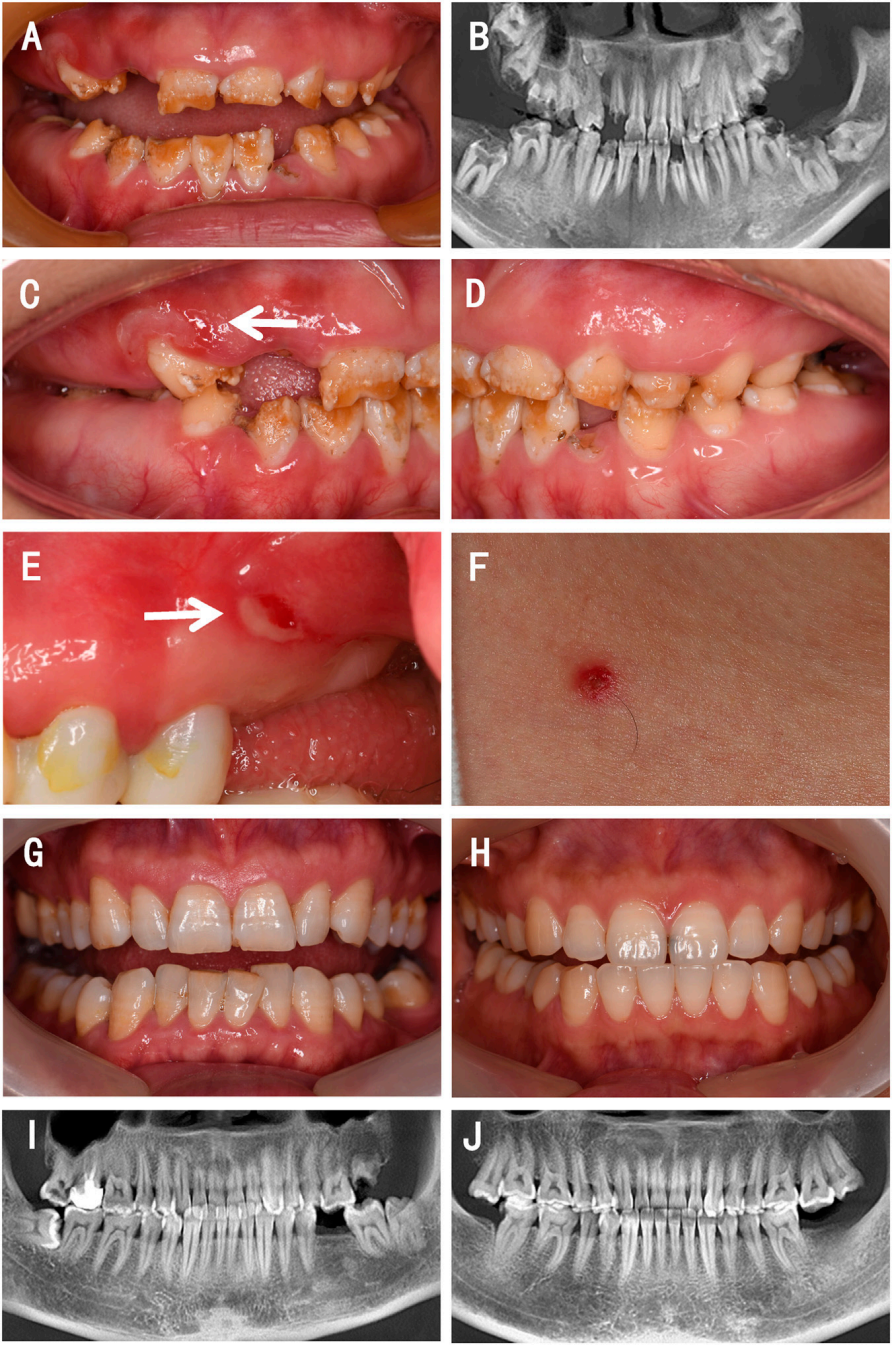
Clinical presentations and radiographic findings of a family with a proband presenting with JEB. **(A,C–E)** Intraoral photographs of the proband. **(B)** Reconstructed panoramic view of the proband’s dentition. **(F)** Photograph of the skin of the proband. **(G)** Intraoral photograph of the proband’s father. **(H)** Intraoral photograph of the proband’s mother. **(I)** Reconstructed panoramic view of the proband’s father. **(J)** Reconstructed panoramic view of the proband’s mother. The white arrows indicate mucosa lesions.

Oral examination of the proband showed that teeth 12, 15, 16, 26, 32, and 45 were residual roots, tooth 63 was retained, and teeth 36 and 46 were decayed. The remaining teeth displayed yellow discoloration with mild-to-moderate attrition. The pits and grooves were evident on the remaining thin, hypoplastic enamel. On some teeth, parts of the enamel were exfoliated, and the underlying dentin was exposed (Figures 1A,C,D). On the panoramic radiograph, teeth 13, 17, 18, 23, 27, 28, 37, 38, and 47 were observed to be impacted, and the enamel of these teeth was extremely thin; however, their radiopacity was still higher than that of the dentin (Figure 1B). Erosion and ulcers were found on the oral mucosa, and a vesicle with clear red liquid appeared on the skin of neck (Figures 1C,E,F).

Her parents showed a normal enamel appearance and structure (Figures 1G–J) and had no mucosal or skin lesions. They denied consanguineous marriage or any abnormal events during pregnancy.

### 3.2 Variants analyses

**FIGURE 2:**
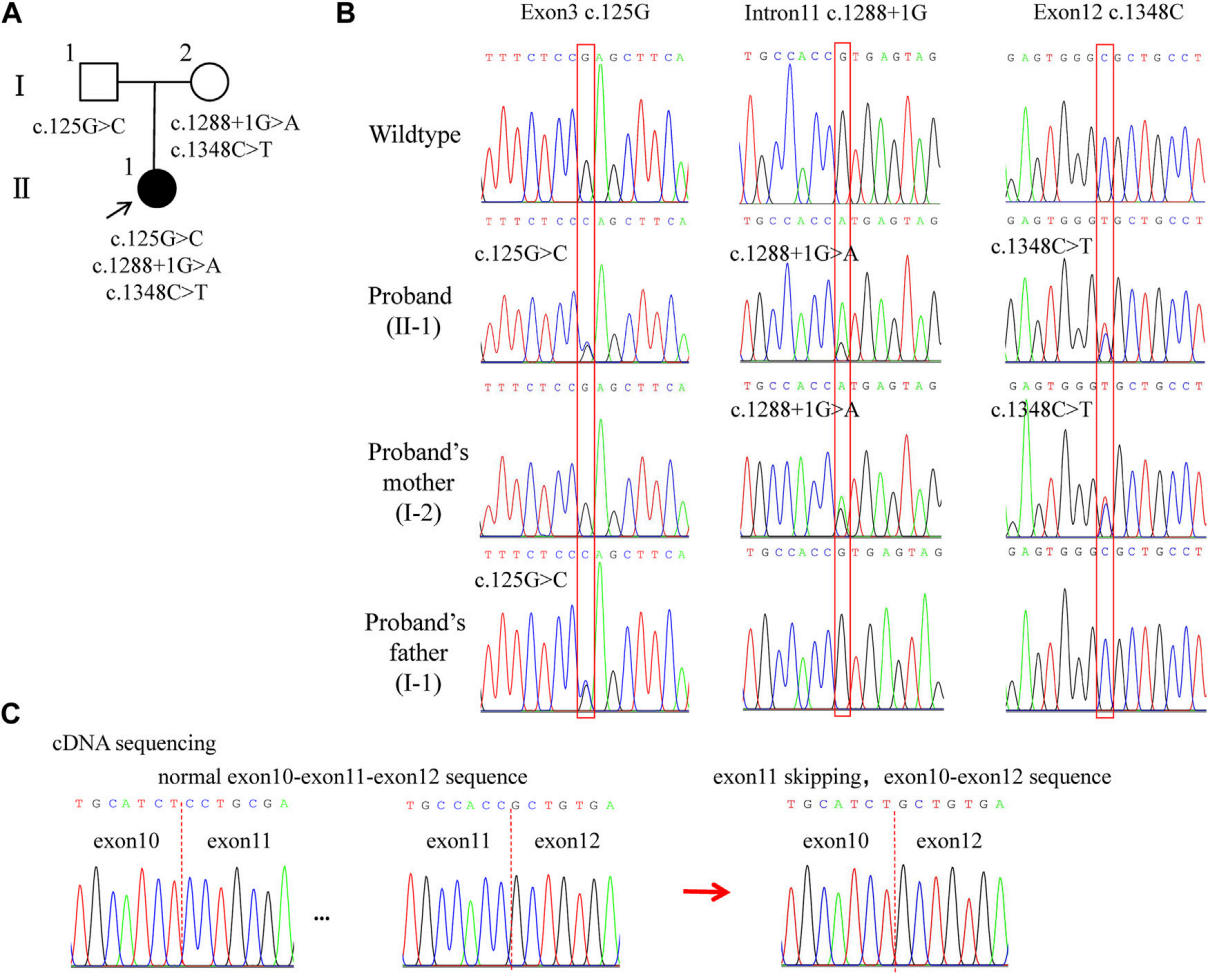
Pedigree and *LAMB3* variants of the family. **(A)** Family pedigree. The black arrow indi-cates the proband. The solid black circle represents the patient. **(B)** Chromatograms showing heterozygous variants: c.125G>C, c.1288+1G>A, and c.1348C>T. **(C)** cDNA sequencing results showing the skipping of exon 11 after splicing. Red boxes indicate the mutational positions. Red dashed lines indicate the boundaries between exons.

**FIGURE 3:**
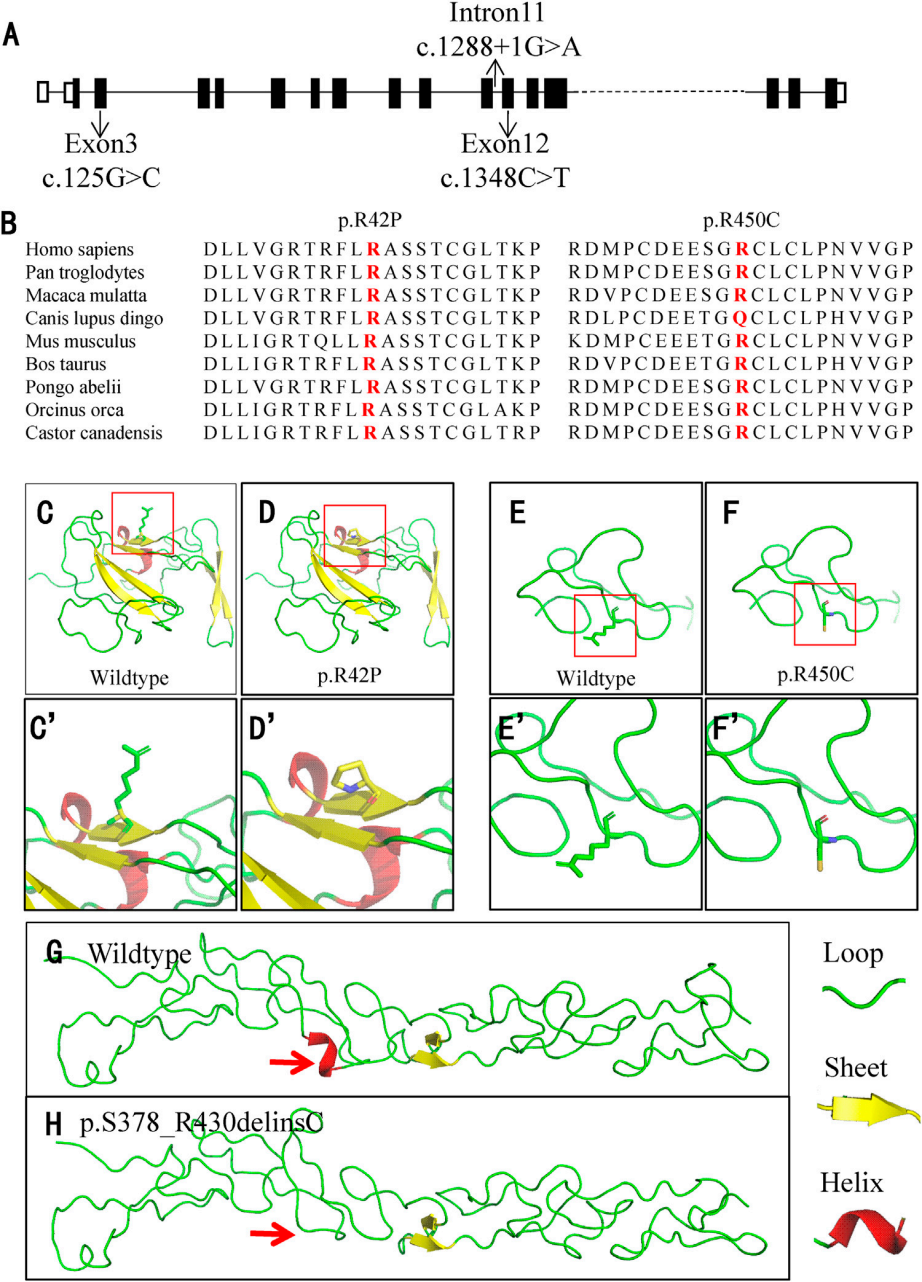
Location and conservation analysis of the variants and tertiary structure analysis of the mutant LAMB3 protein. **(A)** Schematic diagram of *LAMB3* and distribution of the variants. **(B)** Conservation analysis of the affected amino acid in LAMB3 across nine different species. **(C–H)** Analysis of the tertiary structure in wildtype and mutant LAMB3. The red boxes in **(C–F)** indicate the position of changes, and they are amplified in **(C’-F’)**, respectively. Red arrows indicate the differences between the wildtype and mutant protein.

WES revealed compound heterozygous variants of *LAMB3* in the proband: c.125G>C in exon 3, c.1288 + 1G>A in intron 11, and c.1348C>T in exon 12 (part of the variants that were filtered out are shown in Supplementary Table S2). Among these, c.125G>C was inherited from her father, and the other two variants were inherited from her mother (Figures 2A,B, Figure 3A). To explore the effect of the splicing variant c.1288 + 1G>A, we conducted cDNA sequencing, and the result confirmed in-frame skipping of exon 11 after splicing (Figure 2C), which was predicted to result in the deletion of 53 amino acids from serine 378 to arginine 430, and insertion of a cysteine residue (p.S378_R430delinsC).

For the other two missense variants, amino-acid alignment analysis revealed that the two affected residues were highly conserved among different species (Figure 3B). The c.125G>C (p.R42P) variant was predicted to be probably damaging, neutral, and disease-causing in PolyPhen2, PROVEAN, and MutationTaster, respectively. The c.1348C>T (p.R450C) variant was predicted to be probably damaging, deleterious, and disease-causing in PolyPhen2, PROVEAN, and MutationTaster, respectively. PyMOL modeling demonstrated that these variants led to changes in the tertiary structure of LAMB3 (Figures 3C–H). p. R42P variant caused hydrophilic Arg, which has a positively charged side chain (Figures 3C–C’), being substituted with a Pro, which has a rigid side chain restricting the conformation of the protein at this point (Figures 3D–D’). p. R450C variant led to the hydrophilic residue Arg, with a positively charged side chain (Figures 3E–E’) being substituted with a Cys, which has a side chain capable of forming a disulphide bond with another cysteine (Figures 3F–F’). p. S378_R430delinsC variant resulted in a conformational change of a helix loss (Figure 3H), compared to the wildtype (Figure 3G). A summary of the three variants and the predicted results of their functional changes are presented in Table 1.

**TABLE 1:** Summary of the patient’s three *LAMB3* variants and predicted functional change results.

Position	Nucleotide/Protein change	Variant/Hereditary	MutationTaster	PROVEAN	PolyPhen-2	ExAC	ACMG classification (evidence of pathogenicity)
Exon 3	c.125G>C/p.R42P	Missense/parental	Disease-causing	−1.82	0.523	Not present	Likely pathogenic
				Neutral	Probably damaging		PM2 + PM3 + *p*P3 + *p*P4
Intron 11	c.1288 + 1G>A/p.S378_R430delinsC	Splicing/maternal	—	—	—	Not present	Pathogenic
							PVS1 + PM2 + PM4 + *p*P4
Exon 12	c.1348C>T/p.R450C	Missense/maternal	Disease-causing	−3.4	0.984	0.000199	Uncertain significance
				Deleterious	Probably damaging		PM2 + *p*P3 + BP2 + *p*P4

Note: —, not available; ACMG, american college of medical genetics and genomics.

### 3.3 Histological findings of the affected teeth

**FIGURE 4:**
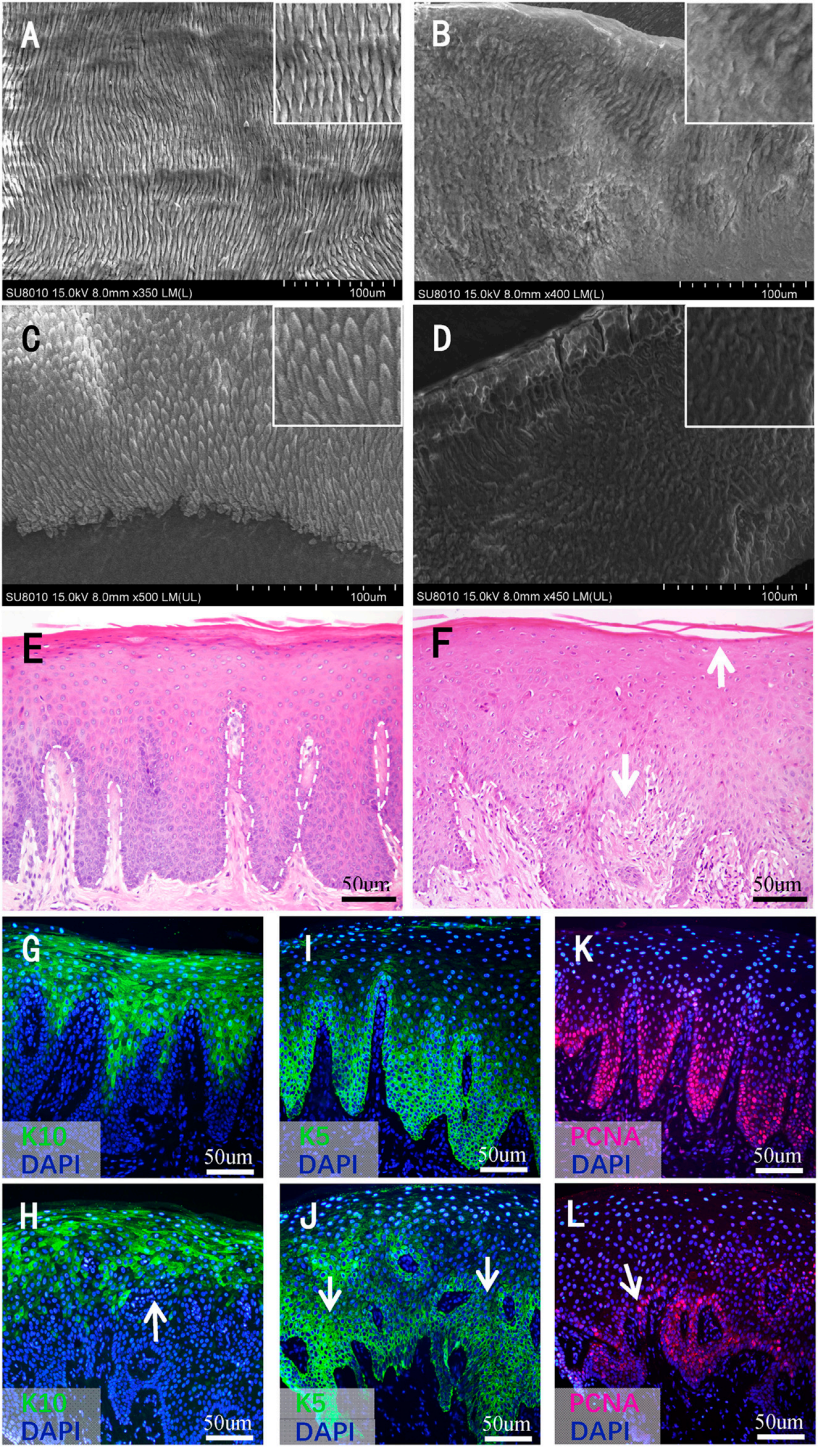
Histological findings of teeth and gingival mucosa. **(A–D)** Scanning electron microscopy images of the teeth. **(A)** The enamel of a normal permanent incisor and **(B)** affected tooth 32. **(C)** The enamel of a normal primary tooth and **(D)** affected tooth 63. Higher magnifications of dentinal tubules are shown in the inset. **(E)** Hematoxylin–eosin staining images of normal gingival mucosa and **(F)** the patient’s mucosa. The white dashed line indicates the boundary between the basal layer and the underlying lamina propria. The white arrows in **(F)** indicate the broken keratinized layer and the blurry boundary between the basal layer and lamina propria, respectively. **(G–L)** Immunofluorescence (IF) staining images of normal gingival mucosa and the patient’s mucosa. **(G)** IF staining of K10 of normal mucosa and **(H)** the patient’s mucosa. **(I)** IF staining of K5 of normal mucosa and **(J)** the patient’s mucosa. **(K)** IF staining of PCNA of normal mucosa and (L) the pa-tient’s mucosa. The white arrows in **(H–L)** indicate the disperse and irregular expression range. meish.

Under SEM, the enamel of the control teeth exhibited a classic prismatic architecture with a well-organized distinct rod appearance on both the permanent (Figure 4A) and the primary (Figure 4C) teeth. However, the enamel of the patient’s permanent (Figure 4B) and primary (Figure 4D) teeth showed a highly rough, amorphous appearance; the prisms were curved and difficult to distinguish, and the rod boundaries were severely blurred and difficult to identify.

### 3.4 Histological findings of the affected gingival mucosa

The mucosa of the control group showed a pattern typical of keratinized epithelium. The keratinized layer was intact and uniform in appearance. The cells in each layer were arranged regularly and stratified. The basal cells settled densely, and the boundary between the basal layer and underlying lamina propria was clear (Figure 4E). As for the mucosa of the patient, the keratinized layer seemed to break off easily. The cells in the granular and prickle layers were not in order. In addition, some of the basal cells were sparsely arranged, and the boundary between the basal layer and lamina propria was not clear in some regions (Figure 4F).

### 3.5 Immunofluorescence staining findings of the affected gingival mucosa

IF tests showed that K10 was expressed in the superficial layer cells of the epithelium; the expression range was larger, and the boundary was clearer in the normal control (Figure 4G) than in the proband (Figure 4H). As for the expression of K5, the expression range of the patient’s gingival mucosa was sparse, and the expression intensity was not uniform (Figure 4J) compared with that of the normal control (Figure 4I). PCNA was expressed in cells close to the basement membrane, the range was larger, and the intensity of expression was stronger in the normal mucosa (Figure 4K) than in the mucosa of the patient (Figure 4L). Compared with the normal mucosa, the expression range of K10, K5, and PCNA in the patient’s mucosa showed a dispersed and irregular outline (Figures 4H,I,L).

## 4 Discussion

*LAMB3* has 23 exons and encodes 1,172 amino acids. It associates with LAMA3 and LAMC2 to form the laminin-332 heterotrimer, which is a component of hemidesmosomes that takes part in the connection between cells and extracellular matrices (Matsui et al., 1995). In the case of the skin, laminin-332 is critical for dermal-epidermal adhesion and can help provide resistance against surface friction (Colognato and Yurchenco, 2000). In the process of enamel formation, laminin-332 was found to be expressed by ameloblast-facing pre-dentine and ameloblasts during the secretory and maturation stages; therefore, laminin-332 was thought to be related to ameloblast differentiation and enamel formation (Yoshiba et al., 1998).

*LAMB3* variants can cause isolated AI and JEB; many patients with JEB also present an AI phenotype (Buchroithner et al., 2004; Farooq et al., 2013; Kiritsi et al., 2015). Phenotype and genotype correlations have been extensively studied, but some confusion remains to be resolved. One heterozygous variant of *LAMB3* causes hypoplastic AI; most of the variants occur in the last two exons (Kim et al., 2013; Kim et al., 2019; Kim et al., 2016; Lee et al., 2015; Poulter et al., 2014; Smith et al., 2019; Wang et al., 2015) except in two cases (Prasad et al., 2016). The variants have been identified as nonsense or frameshift mutations, all of them resulting in a truncated protein. Presumably, the premature termination codon that occurs in this location probably causes the transcripts to escape nonsense-mediated decay (NMD) and allow the truncated protein to be secreted; hence, the phenotype is confined to the enamel and does not develop into JEB (Lee et al., 2015; Poulter et al., 2014). Biallelic variants of *LAMB3* lead to JEB, and these variants are mostly located in the first 21 exons. The patient in our study inherited her variants in an autosomal recessive pattern, with c.125G>C from her father and c.1288 + 1G>A and c.1348C>T from her mother. In addition to the enamel defects, erosion and ulcers of the oral mucosa and small range of skin lesions were detected; therefore, she was diagnosed with intermediate JEB. The c.125G>C and c.1348C>T mutations were predicted to be disease-causing according to several prediction algorithms. PyMOL modeling demonstrated that the variants led to changes in the tertiary structure of LAMB3. These changes may lead to a decrease of protein stability. We obtained a gingival sample from the patient, and subsequent cDNA sequencing showed that variant c.1288 + 1G>A resulted in exon 11 skipping during splicing. The missense and splicing variants caused amino-acid substitutions and in-frame deletions, but did not cause severely truncated proteins or downstream frameshift, which may be the reason for why the proband’s parents did not present apparent defects in their enamel.

The enamel in patients with a single-locus *LAMB3* variant is reported to have generalized, irregular pitting and grooves; some patients also showed severely deformed molar crowns (Smith et al., 2019; Wang et al., 2015). The enamel in JEB cases in which the patients have biallelic *LAMB3* variants has been described as hypoplastic enamel with decay (Buchroithner et al., 2004), although, in many JEB cases, the teeth were not examined. In our case, pits and grooves were obvious on the remaining thin, hypoplastic enamel; enamel exfoliation and dentin exposure could be observed on some teeth, and subsequent decay and residual roots were easily found. However, crown malformations such as excess cusps were not observed. It has been suggested that the C-terminal domain of LAMB3 may be essential for proper enamel cusp formation (Smith et al., 2019); thus, different variant locations could lead to different crown appearances. In another study, SEM examination of the third molar showed that the enamel structure was abnormal near the EDJ (Smith et al., 2019). In our case, the enamel of the permanent tooth was rough and amorphous, the prisms were curved and difficult to distinguish, and abnormality could be observed along the whole layer of enamel. This may be due to different variant loci. In the aforementioned study, the variant was on a single allele and occurred in the last exon, which allowed it to escape NMD. In our study, the patient had biallelic variants, and the three variants were all predicted to be pathogenic; therefore, the pathological changes in the enamel were more severe. In addition, this is the first study to report the microstructure of the primary tooth in *LAMB3*-related patients. The prisms of the primary tooth were curved along the whole layer, but the boundaries between prisms were clearer than those of the permanent teeth. Further studies are needed to confirm this finding and to identify the distinct functional role of *LAMB3* in the amelogenesis of primary and permanent teeth.

Because JEB is a disease characterized by congenital skin and mucosal blistering, there have been many studies on the skin. On perilesional skin, splits were observed along the lamina lucida (Mayer et al., 2016), and IF staining of the laminin β3 chain showed reduced or absent expression (Kiritsi et al., 2015; Mayer et al., 2016). However, to date, there have been no reports of histological examination of the oral mucosa. In our study, HE staining showed that the affected mucosa had an impaired keratinized layer and misaligned cell arrangement. In addition, the boundary between the basal layer and lamina propria was not as clear as in the control group. IF staining of K10, K5, and PCNA showed that the expression range in the patient’s mucosa had a dispersed and irregular outline; the expression range of PCNA was larger and its intensity was stronger in the control mucosa than in the patient’s mucosa. These results indicate impaired protective and proliferative function of the epithelium, which may explain the appearance of ulcers and erosion of the patient’s mucosa.

In summary, we identified rare compound heterozygous variants of *LAMB3* in a patient with intermediate JEB, which extend the variant spectrum of the *LAMB3* gene and JEB cases. Our findings provide new evidence related to genotype-phenotype correlation of the disease. Most importantly, this is the first study to identify histological malformations of the primary teeth and oral mucosa in *LAMB3-*related patients. However, the precise role of *LAMB3* in tooth and mucocutaneous development remains to be discovered in further studies.

## Data Availability

The datasets presented in this study can be found in online repositories. The names of the repository/repositories and accession number(s) can be found in the article/Supplementary Material.
