# Supplementary material for: Rare compound heterozygous variants of LAMB3 and histological features of enamel and oral mucosa
Source: Front Physiol. 2022 Oct 10;13:1006980. doi: 10.3389/fphys.2022.1006980 (PMC9589216; doi:10.3389/fphys.2022.1006980)
Supplement: Supplementary file 2 [file Table1.DOCX]

**Table S1** Primers used in *LAMB3* amplification.

| Name | Product(bp) | Forward primer | Reverse primer |
| --- | --- | --- | --- |
| Exon 3 | 151 | CTGTGATTTCAGCCCTGCC | CAGTAGGTCTCAGGCTTGGT |
| [Intron 11](http://asia.ensembl.org/Homo_sapiens/Location/View?db=core;g=ENSG00000196878;r=1:209627530-209628084;t=ENST00000356082) | 672 | AGTGTGTGTGCAAGGAGCAT | CAGTGGTAGGGAGCACACTG |
| Exon 12 | 210 | CTGTGACTGCAACATCCTGG | CCCTCACTTCGTACCTGGTT |
| cDNA | 917 | CTGCTGTTGTCTCTCAGCCT | CCTAGAAGGGCAGCATGGAG |
